# Molecular epidemiology of HIV-1 strains in Antalya, Turkey

**DOI:** 10.7448/IAS.17.4.19684

**Published:** 2014-11-02

**Authors:** Dilara Inan, Murat Sayan

**Affiliations:** 1 Infectious Diseases and Clinical Microbiology, Faculty of Medicine, Akdeniz University Hospital, Akdeniz University, Antalya, Turkey; 2 Clinical Laboratory, PCR Unit, Kocaeli University Hospital, Kocaeli University, Izmit-Kocaeli, Turkey

## Abstract

**Introduction:**

The aim of this study was to determine the subtype distribution of HIV-1 strains isolated from antiretroviral therapy (ART)-naive and on treatment patients in Antalya, the city of southern Turkey.

**Materials and Methods:**

The study included 77 of 92 newly diagnosed HIV-1 positive patients of last two years (between February 2012 and June 2014). HIV-1 subtypes and circulating recombinant forms (CRFs) were identified by phylogenetic analysis of reverse-transcriptase (codon 41–238) and protease (codon 1–99) domains (~667 bp) of *pol* gene region in HIV-1 strains.

**Results:**

Subtype B (48%, 37/77) was identified as the most common HIV-1 subtype in Antalya that similar the other Turkish patients. Non-B subtypes were followed as CRFs (39%, 30/77). Interestingly, CRF14_BG (12.9%, 10/77) was found for the first time in Antalya in contrast to previous observations in the other reports in Turkey. Also, subtype G (6.5%, 5/77) was detected more often than HIV-1 subtypes in circulation of Turkey.

**Conclusions:**

These findings may be associated with specific geographic localization of Antalya that touristic movements of city. Recognized HIV-1 subtype diversity is major challenges in the development of a globally effective HIV vaccine.
